# A quantitative analysis of bestrophin 1 cellular localization in mouse cerebral cortex

**DOI:** 10.1111/apha.14245

**Published:** 2024-10-28

**Authors:** Michael Di Palma, Wuhyun Koh, C. Justin Lee, Fiorenzo Conti

**Affiliations:** ^1^ Department of Experimental and Clinical Medicine, Section of Neuroscience and Cell Biology Università Politecnica delle Marche Ancona Italy; ^2^ Center for Cognition and Sociality Institute for Basic Science Daejeon South Korea; ^3^ Center for Neurobiology of Aging IRCCS INRCA Ancona Italy

**Keywords:** astrocytes, BEST1, cerebral cortex, microglia, neurons, oligodendroglia

## Abstract

**Aim:**

Calcium‐activated ligand‐gated chloride channels, beyond their role in maintaining anion homeostasis, modulate neuronal excitability by facilitating nonvesicular neurotransmitter release. BEST1, a key member of this family, is permeable to γ‐aminobutyric acid (GABA) and glutamate. While astrocytic BEST1 is well‐studied and known to regulate neurotransmitter levels, its distribution and role in other brain cell types remain unclear. This study aimed to reassess the localization of BEST1 in the mouse cerebral cortex.

**Methods:**

We examined the localization and distribution of BEST1 in the mouse parietal cortex using light microscopy, confocal double‐labeling with markers for astrocytes, neurons, microglia, and oligodendrocyte precursor cells, and 3D reconstruction techniques.

**Results:**

In the cerebral cortex, BEST1 is more broadly distributed than previously thought. Neurons are the second most abundant BEST1^+^ cell type in the cerebral cortex, following astrocytes. BEST1 is diffusely expressed in neuronal somatic and neuropilar domains and is present at glutamatergic and GABAergic terminals, with a prevalence at GABAergic terminals. We also confirmed that BEST1 is expressed in cortical microglia and identified it in oligodendrocyte precursor cells, albeit to a lesser extent.

**Conclusions:**

Together, these findings suggest that BEST1's role in controlling neurotransmission may extend beyond astrocytes to include other brain cells. Understanding BEST1's function in these cells could offer new insights into the molecular mechanisms shaping cortical circuitry. Further research is needed to clarify the diverse roles of BEST1 in both normal and pathophysiological conditions.

## 1 INTRODUCTION

Bestrophins are a family of ligand‐gated chloride (Cl^−^) channels activated by calcium (Ca^2+^). They were identified by linkage to hereditary macular degeneration caused by mutations in one of the four known paralogues in mammals, bestrophin 1 (BEST1).^1^, ^2^ These channels exhibit a homo‐pentameric structure with two significant occlusions to the ion conduction pathway along the channel axis: the neck and aperture, which contribute to anion selectivity and gating.^3^, ^4^, ^5^ Beyond its contribution to anion homeostasis,^6^, ^7^ BEST1 reportedly contributes to other physiological processes, including cell volume regulation,^8^, ^9^ pH homeostasis,^10^ and neurotransmitter release.^11^ Despite conflicting structural data on the pore dimension,^5^, ^12^, ^13^ functional evidence demonstrated the permeability of BEST1 for large anions, γ‐aminobutyric acid (GABA) and glutamate.^11^, ^14^

Early studies showed that BEST1 is predominantly expressed in the human retinal pigment epithelium,^1^, ^2^ while later studies proved that the BEST1 transcripts are broadly distributed in the mouse brain, with higher levels in the olfactory bulb, hippocampus, and cerebellum.^15^, ^16^ BEST1 mRNA was found in acutely dissociated astrocytes and neurons from adult mouse cortex, and the protein expression was immunohistochemically confirmed in both cellular compartments in the mouse hippocampus.^15^ Despite this, most of the research on this anion channel has primarily focused on astrocytic localization and function, leaving its role and distribution in other cellular compartments largely unexplored. Indeed, astrocytic BEST1 mediates nonvesicular GABA and glutamate release, influencing synaptic transmission and plasticity,^16^, ^17^, ^18^ whereas critical information on its localization beyond the astrocytic domain still needs to be clarified. Recent evidence strengthened this concern. It was shown that stroke‐induced extrasynaptic glutamate release through neuronal BEST1 leads to delayed excitotoxicity in the mouse motor cortex^19^ and that cortical microglial cells enhance GABA uptake through the engagement of an interplay between the GABA transporter 1 (GAT‐1) and this channel upon a neuroinflammation insult.^20^ This evidence indicates that BEST1‐mediated regulation of neurotransmission may involve diverse cellular compartments. Therefore, in order to further our understanding of BEST1's role in cortical circuits, we aimed at providing a more comprehensive characterization of BEST1 cellular localization within the mouse cerebral cortex.

## 2 RESULTS

### 2.1 Differential distribution and localization of BEST1 in cerebral cortex

#### 2.1.1 Light microscopy inspection of BEST1 immunoreactivity in mouse parietal cortex

**FIGURE 1:**
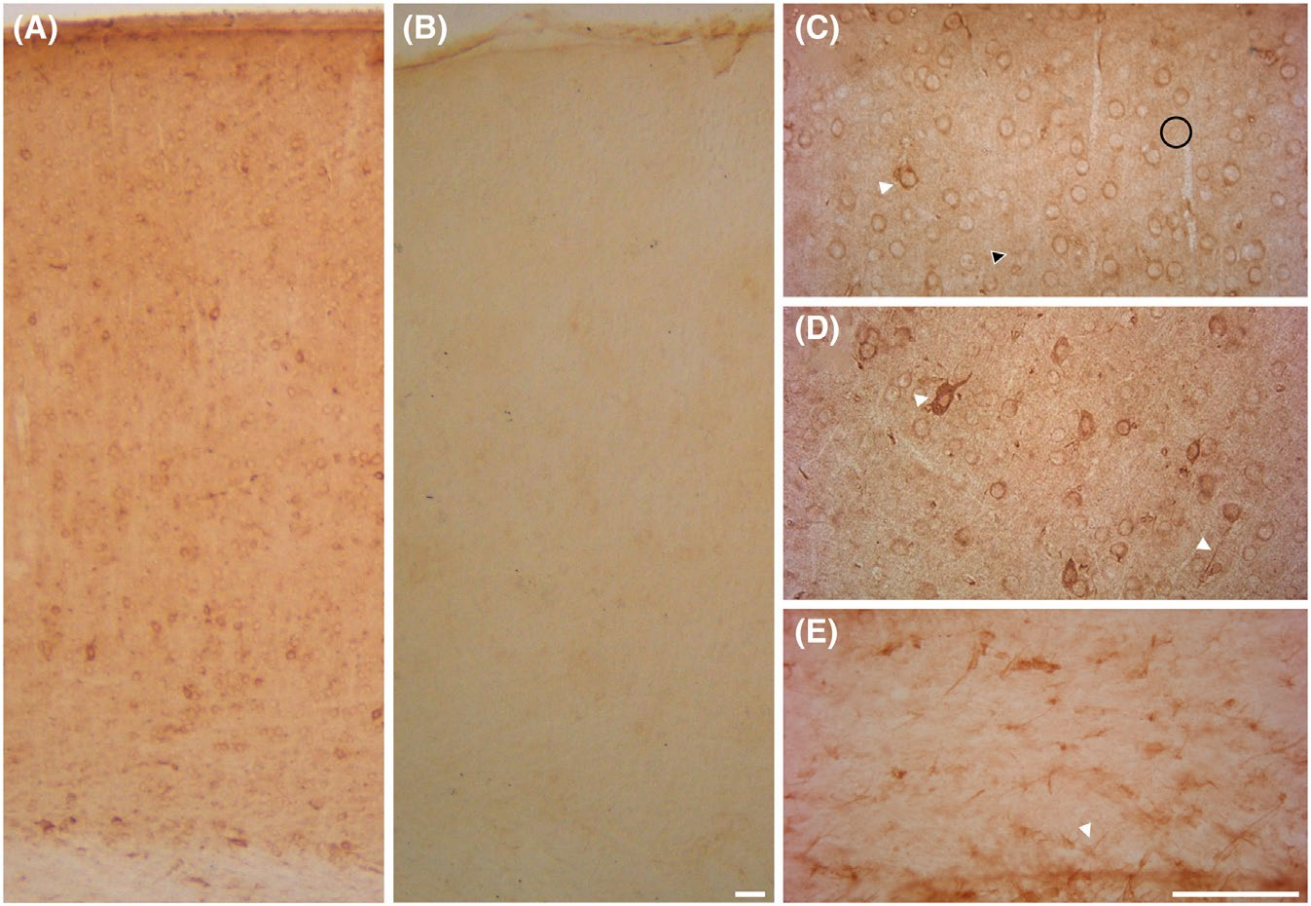
Distribution of BEST1 immunoreactivity (ir) in wild‐type and knockout mice cerebral cortex. (A) Distribution of Best1 across the parietal cortex in wild‐type (WT) and (B) global BEST1 knockout (KO) animals. Cortical fields were acquired at the original magnification of 20×. (C) High magnification fields of cortical layers 2/3, (D) layer 5, and (E) corpus callosum in WT condition. A diffuse neuron‐like cell BEST1 ir (white arrowheads in C and D) across SI cortical layers 2/3 and 5 was detectable. Occasional neuropil elements (black arrowhead in C), faint glial‐like BEST^+^ cells (black circle in C), and BEST1^+^ vertical profiles were also detectable. A diffuse glial‐like Best1^+^ cells were detectable in the corpus callosum (white arrowhead in E). Scale bar: A–B, 80 μm; C–E, 200 μm.

Light microscopy inspection of mouse parietal cortex revealed that BEST1 immunoreactivity (BEST1‐ir) was present in all layers (Figure 1A) and that it was abolished in knockout mice (Figure 1B). BEST1‐ir was mostly in perikarya and proximal processes of neuron‐like cells, and occasional immunoreactive profiles and neuropil elements were also present in the neuropil (Figure 1C,D). Faint BEST1 glial‐like^+^ cells were observed across cortical layers, whereas BEST1‐ir was strong in numerous glial‐like^+^ cells in underlying white matter (Figure 1E).

#### 2.1.2 Cortical neurons represent the second most abundant BEST1 ^+^ cell type in the cerebral cortex

**FIGURE 2:**
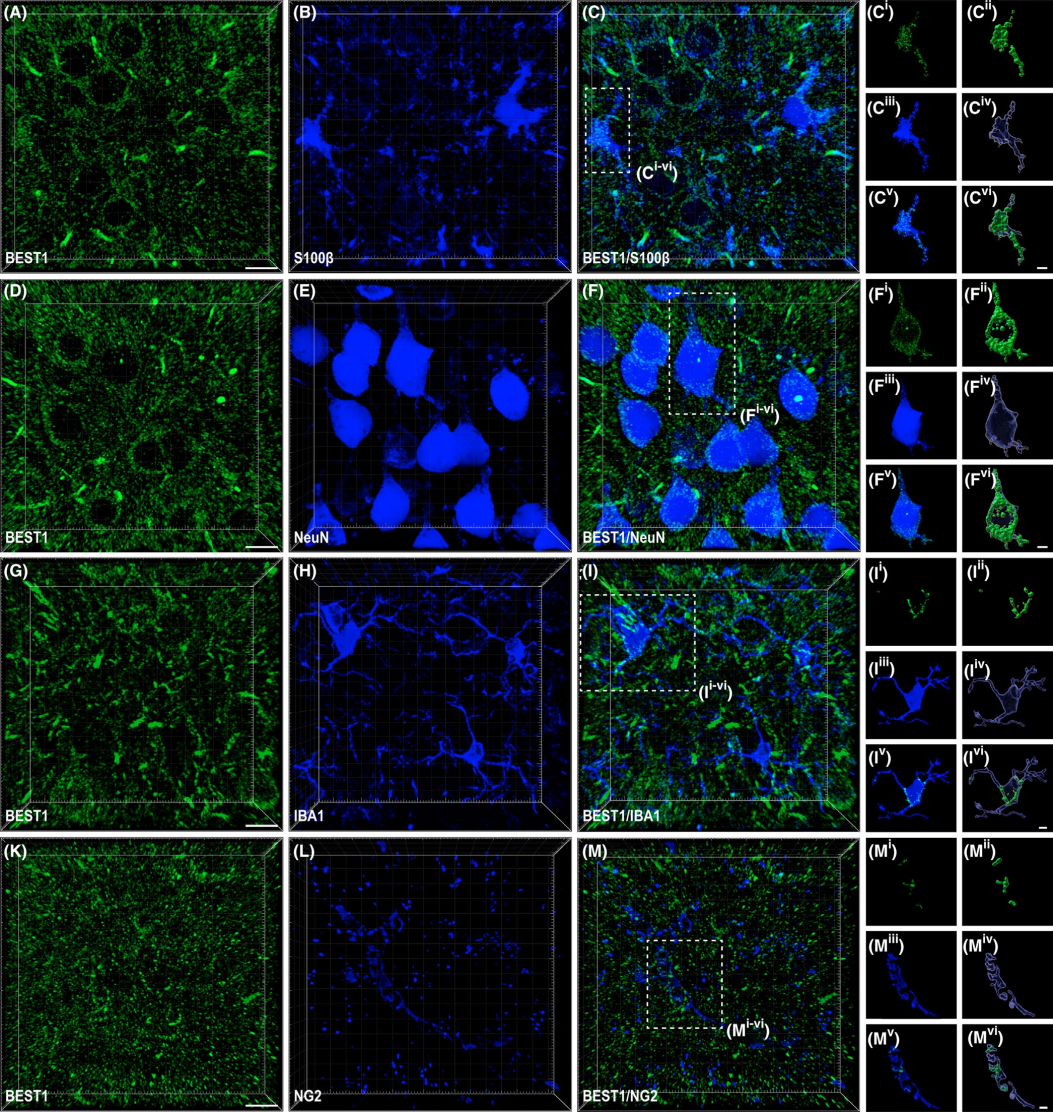
Distribution of BEST1 immunoreactivity (ir) in layers 2/3 of mouse parietal cerebral cortex. (A) Representative confocal 2D single *z*‐stack of BEST1 (green), (B) S100β (blue), and (C) BEST1/S100β overlay. (D) Representative confocal 2D single *z*‐stack of BEST1 (green), (E) NeuN (blue), and (F) BEST1/NeuN overlay. (G) Representative confocal 2D single *z*‐stack of BEST1 (green), (H) IBA1 (blue), and (I) BEST1/IBA1 overlay. (K) Representative confocal 2D single *z*‐stack of BEST1 (green), (L) NG2 (blue), and (M) BEST1/NG2 overlay. Representative 2D image with the respective 3D surface volume reconstructions of BEST1 (C^i–ii^, F^i–ii^, I^i–ii^ and M^i–ii^) and the cellular marker (S100β, C^iii–iv^; NeuN, F^iii–iv^; IBA1, I^iii–iv^, and NG2, M^iii–iv^) staining with their overlay (BEST/S100β, C^v–vi^; BEST/NeuN, F^v–vi^; BEST/IBA1, I^v–vi^, and BEST/NG2, M^v–vi^). Scale bars: A–M, 10 μm; C^i–vi^–M^i–vi^, 4 μm.

**FIGURE 3:**
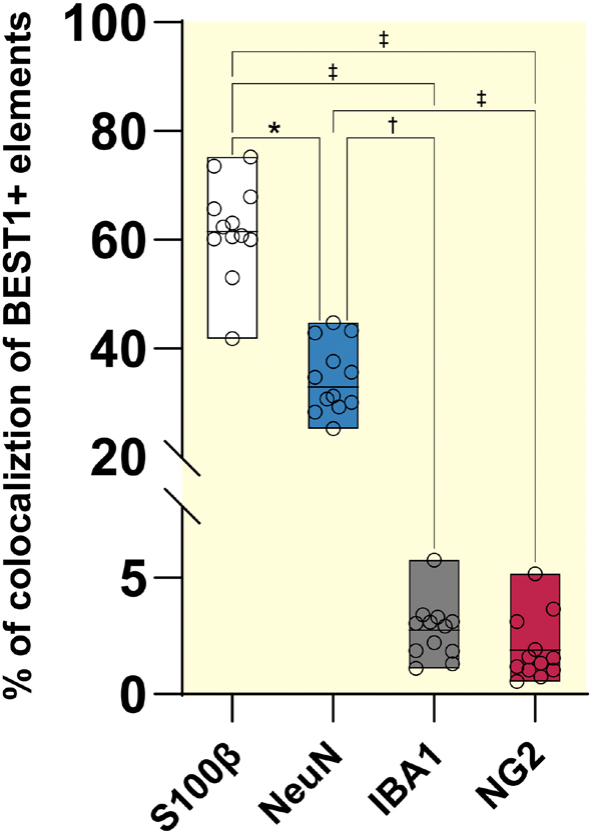
Cellular BEST1 expression comparison in layers 2/3 of mouse parietal cerebral cortex. Percentage of the volume of BEST1^+^ elements colocalized with each cellular marker (Kruskal–Wallis test *χ* ^2^ (3, *N* = 60) = 46.51, *p* < 0.001; Dunn's post hoc test: *p < 0.05; ^†^ *p* < 0.01 and ^‡^ *p* < 0.001). Data are expressed as median ± Min and Max.

To determine the nature of cortical BEST1^+^ cells, we performed double‐labeling studies with BEST1 and astroglial (S100β^21^, ^22^; Figure 2A–C,C^i–vi^), neuronal (NeuN^23^; Figure 2D–F,F^i–vi^), microglial (IBA1^24^; Figure 2G–I,I^i–vi^), or oligodendrocyte precursor cells—OPCs (NG2^25^; Figure 2K–M,M^i–vi^) markers. These analyses were carried out in cortical layers 2/3, which have the highest number of glutamatergic and GABAergic synapses.^26^ BEST^+^ cells were then processed to quantify the colocalization with the BEST1 signal using Imaris 3D surface reconstruction of BEST1 and cellular marker volume^20^ (Figure 2). We confirmed that BEST1 is mainly expressed in astrocytes since we found that, out of ~60 000 μm^3^ of BEST1 volume, more than half (*M* = 61.97%, *SD* ±8.84%) colocalized with S100β^+^ cells (*N*_cells_ = 48; Figure 3). However, we found that 34.40% (*SD* ±5.32%) of the volume of BEST^+^ elements colocalized with NeuN^+^ cells (*N*_cells_ = 48; Figure 3), thus indicating that neurons represent over one‐third of all BEST1^+^ cells. Intriguingly, this channel was localized in the perikarya and proximal processes of NeuN^+^ cells (Figure 2F^i–vi^), suggesting a potential differential subcellular distribution between extrasynaptic and synaptic domains at the neuronal level. Even if with significantly lower levels compared to astrocytes and neurons, we consistently found that BEST1 was colocalized with IBA^+^ cells (*N*_cells_ = 48; *Md* = 2.97%, *SD* ±1.23%; Figure 3). A similar amount of colocalization to that found in IBA^+^ cells was obtained in NG2^+^ (*N*_cells_ = 48; *Md* = 1.90%, *SD* ±1.38%; Figure 3), thus revealing that the BEST1 channel is expressed even in OPCs (for details on antibodies, see Table 1).

**TABLE 1:** Primary and secondary antibodies.

Primary antibodies					
Antibodies	Host	Dilution	Source	Characterization	Identifier
BEST1	R	1:5000 (Ip) 1:2000 (If)	Kindly provided by Dr. Justin Lee (Center for Cognition and Sociality, Institute for Basic Science, Daejeon, South Korea)	Park et al. (2009)^15^; Kwak et al. (2020)^45^	N.A.
S100β	GP	1:200 (If)	Synaptic system	Dorst et al. (2021)^46^; Lia et al. (2023)^47^	Cat # 287 004, RRID:AB_2620025
NeuN	GP	1:200 (If)	Synaptic system	Nott et al. (2016)^48^; Grossman et al. (2017)^49^	Cat # 266 014, RRID:AB_2924930
IBA1	GP	1:500 (If)	Synaptic system	Kleidonas et al. (2023)^50^; Yamasaki et al. (2024)^51^	Cat # 234 308, RRID:AB_2924932
NG2	M	1:200 (If)	Millipore	Goncalves et al., 2019^52^; Corkrum et al. (2020)^53^	Cat # 05‐710, RRID:AB_309925
VGLUT1	GP	1:500 (If)	Synaptic system	Ricciardi et al. (2012)^54^; Souter et al. (2021)^55^	Cat # 135 318, RRID:AB_2924948
VGAT	GP	1:500 (If)	Synaptic system	Wang et al. (2021)^56^; Kim et al. (2023)^57^	Cat # 131 005, RRID:AB_1106810

Secondary antibodies				
Conjugated to	React	Dilution	Source	Identifier
Biotinylated	R	1:250 (Ip)	Jackson ImmunoResearch	Cat # 711‐066‐152, RRID:AB_2340594
Alexa Fluor 488®	R	1:300 (If)	Jackson ImmunoResearch	Cat # 711‐546‐152, RRID:AB_2340619
Alexa Fluor 647®	GP	1:300 (If)	Jackson ImmunoResearch	Cat # 706‐606‐148, RRID:AB_2340477

Abbreviations: GP, guinea pig; If, immunofluorescence; Ip, immunoperoxidase; M, mouse; R, rabbit.

These data demonstrate a broader cellular distribution of BEST1 expression at the cortical level. In addition, they indicate that neurons represent the second most abundant BEST1^+^ cell type in the cerebral cortex.

### 2.2 BEST1 is expressed at presynaptic domains

#### 2.2.1 BEST1 is localized at both glutamatergic and GABAergic terminals, with higher prevalence at GABAergic terminals

**FIGURE 4:**
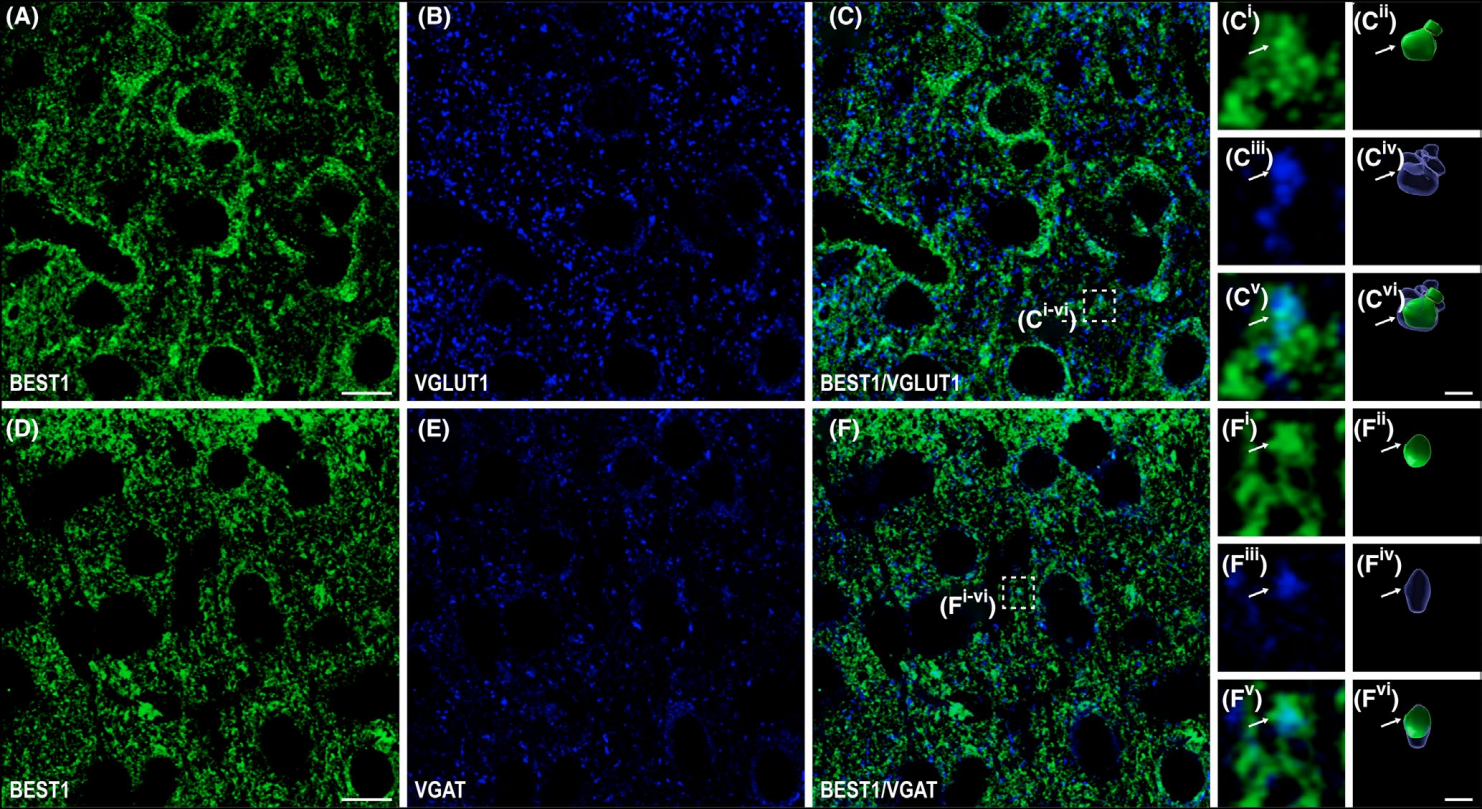
BEST1 distribution at glutamatergic and GABAergic presynaptic domains in layers 2/3 of mouse parietal cortex. (A) Representative confocal 2D single *z*‐stack of BEST1 (green), (B) VGLUT1 (blue), and (C) BEST1/VGLUT1 overlay. (D) Representative confocal 2D single *z*‐stack of BEST1 (green), (E) VGAT (blue), and (F) BEST1/VGAT overlay. High magnification images of neuropilar BEST1^+^ elements (C^i^ and F^i^, 3D surface reconstruction in C^ii^ and F^ii^) and the synaptic marker (VGLUT1^+^ puncta in C^iii^ and VGAT^+^ puncta in F^iii^, 3D surface reconstruction of VGLUT1^+^ puncta in C^vi^ and VGAT^+^ puncta in F^iv^). Puncta were considered double‐labeled (arrow in C^v^ and F^v^) when physically overlapped (3D surface reconstruction in C^vi^ and F^vi^). Scale bars A–C and D–F, 10 μm; C^i–vi^ and F^i–vi^, 0.5 μm.

**FIGURE 5:**
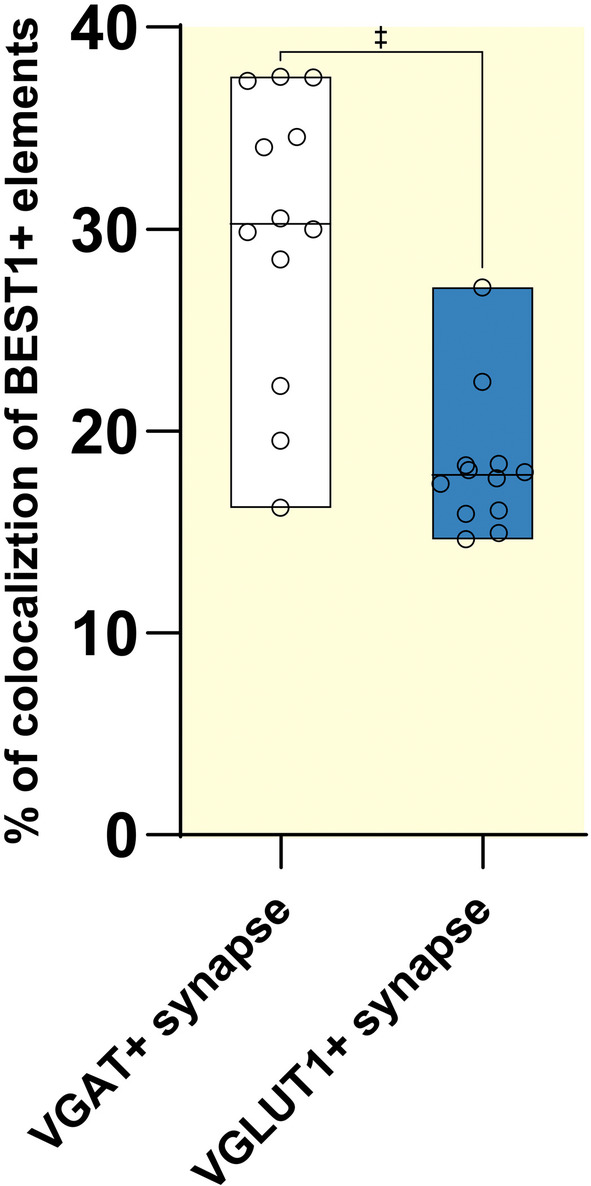
Presynaptic BEST1 expression comparison in layers 2/3 of mouse parietal cerebral cortex. Percentage of BEST1^+^/VGAT^+^ versus BEST1^+^/VGLUT^+^ colocalized elements (Mann–Whitney *U* = 12, ^‡^ *p* < 0.001, *n* = 12 for each group). Data are expressed as median ± Min and Max.

BEST1 distribution at the neuronal level was further explored to determine the nature of BEST1 expressing neurons and whether its expression is pre‐ or postsynaptic. To this end, we studied the colocalization of BEST1^+^ elements with glutamatergic (VGLUT1^27^; Figure 4A–C,C^i–vi^) or GABAergic (VGAT^28^; Figure 4D–F,F^i–vi^) markers. BEST1^+^/VGLUT1^+^ and BEST1^+^/VGAT^+^ colocalization analyses were carried out 1–3 μm beneath the section surface.^29^ This analysis confirmed the neuropilar expression of BEST1 and showed a significantly higher content of BEST1 at GABAergic than at glutamatergic synapses (*M*_BEST1+/VGAT1+ puncta_ = 29.82%, *SD* ±7.18% vs. *M*_BEST1+/VGLUT+ puncta_ = 18.24%, *SD* ±3.45%; Figure 5). For details on antibodies, see Table 1. These findings collectively reveal that BEST1 is expressed presynaptically in the cerebral cortex, implying that this channel may modulate neurotransmitter release at the synaptic level.

## 3 DISCUSSION

Our findings reveal that, at least in cerebral cortex, the BEST1 channel has a broader cellular distribution than previously thought. Neurons are the second most abundant BEST1^+^ cell type in the cerebral cortex, following astrocytes. The channel is diffusely expressed in neuronal somatic and neuropilar domains and is localized at both GABAergic and glutamatergic presynaptic terminals. Additionally, our results show that microglia and oligodendrocyte precursor cells (OPCs) also express BEST1, providing a more comprehensive understanding of its distribution and challenging the predominant view that its functional role is mediated almost exclusively by astrocytes.

Recent findings showed that stroke‐induced extrasynaptic glutamate release via BEST1 leads to delayed excitotoxicity in the mouse motor cortex,^19^ suggesting that BEST1 may play a role in modulating neuronal slow tonic neurotransmitter release in pathological conditions. Our demonstration of neuronal BEST1 expression in perikarya and proximal processes of cortical neurons suggests that this assumption may be extended to physiological conditions. Glutamate and GABA tone have been shown to affect the network activity of cortical neurons and information processing.^30^, ^31^, ^32^, ^33^, ^34^ Compelling evidence proved that BEST1‐mediated gliotransmission primarily controls both GABA and glutamate tone in physiological and pathological conditions.^17^, ^18^ By demonstrating that over one‐third of the cortical BEST1 channel is expressed in neuronal cells, we can postulate a novel mechanism through which glutamate and GABA tone regulation may partially be accounted for by direct neuronal action via BEST1. Our analyses also reveal, for the first time, the presynaptic expression of BEST1 at both glutamatergic and GABAergic terminals, with higher content at GABAergic terminals. This finding suggests that BEST1 may control neurotransmission directly in the synaptic domain. Recently, Romei et al. found that in synaptosomes, GABA uptake by GABA transporter 1 (GAT‐1) induces GABA release that is in part mediated by an anion channel.^35^ BEST1 could be this channel, suggesting that an interplay between GABA transporters and BEST1 may occur in controlling synaptic GABA fine‐tuning within the parietal cortex. It appears that investigating the role of neuronal BEST1 could shed light on the molecular underpinnings of critical events shaping cortical circuitry. Such studies employing genetic, as astrocyte‐specific BEST1 conditional knockout strains,^36^ or optogenetic tools^37^ could clarify the temporal dynamics of BEST1‐mediated neurotransmitter release and its impact on higher‐order functions like information processing, sensory discrimination, and perceptual learning. Additionally, since GABA and glutamate level tuning play a pivotal role in the pathophysiology of several neurological and psychiatric disorders, understanding these dynamics might reveal novel therapeutic strategies. Notably, both cognitive disorders, such as Alzheimer's and Parkinson's diseases,^17^ and psychiatric conditions, including schizophrenia and autism spectrum disorder,^18^ exhibit abnormal GABA and glutamate tone. In this complex context, neuronal BEST1 may serve as an additional pharmacological target for treating these disorders.

Finally, we showed that microglia and OPCs express the BEST1 channel in the mouse parietal cortex. In 2023, we demonstrated that in primary microglial cells dissociated by mice cortices, a novel functional interplay between GAT‐1 and BEST1 occurs in inflammatory conditions, and it is critical in modulating microglial GABA clearance.^20^ Our analyses confirmed that BEST1 is localized in cortical microglial cells and extend previous findings by revealing that this channel is constitutively expressed in these cells in physiological conditions. Recently, OPCs were shown not only to contain GABA but also to play a role in mediating tonic inhibition and fine‐tuning synaptic transmission.^38^ This research found that OPCs express glutamate decarboxylase 67, suggesting that this enzyme synthesizes GABA within these cells.^38^ Besides de novo synthesis, these cells may uptake this neurotransmitter by the extracellular milieu. In keeping with this, we showed that both OPCs and mature oligodendroglia express GAT‐1 and mediate a GAT‐dependent GABA uptake.^39^ Here, we showed that BEST1, although at very low levels, is localized in oligodendrocyte precursor cells (OPCs) in the mouse parietal cortex. This finding suggests that, besides synthesizing and taking up GABA, these cells may also release this neurotransmitter, potentially influencing circuitry transmission. Given this evidence, it remains to be explored whether BEST1‐mediated nonvesicular GABA release in oligodendroglia regulates GABA tone to mediate tonic inhibition. Future research in this area is essential for advancing our understanding of the mechanisms underlying higher‐order cognitive functions and for identifying new pharmacological targets for various neurological disorders associated with oligodendroglia disfunction.

## 4 MATERIALS AND METHODS

### 4.1 Animals and tissue preparation

All mice were kept in a temperature‐ and humidity‐controlled environment with a 12‐h light–dark cycle (lights on at 7 a.m.) and had free access to food and water. All animal care and handling were approved by the local animal research ethics committee and performed according to the European Union legislation (Directive 2010/63/EU) and the Italian law on animal experimentation (D.lgs 26/2014; research project permitted with authorization N° 40A31.N.ZUK by the Italian Ministry of Health). Two‐month‐old male C57BL6 wild‐type (WT) mice (*N* = 24) were used for immunohistochemical procedures. After induction of deep anesthesia with chloral hydrate (12%; i.p.), animals were perfused through the ascending aorta with physiological saline followed by 4% paraformaldehyde (PFA) in 0.1 M phosphate buffer (PB; pH 7.4). The brains were removed and postfixed in the same fixative at 4°C until processed for histology. For immunoperoxidase control experiments, brain samples, processed as described above, from male C57BL/6JCya‐*Best1* ^*em1*^ (BEST1 global knockout; MGI # 1346332, *N* = 5) and their WT (*N* = 5) littermate mice were kindly provided by Dr. Justin Lee (Center for Cognition and Sociality, Institute for Basic Science, Daejeon, South Korea).

#### 4.1.1 Antibodies

The appropriate working dilution for each antibody was determined using both previously published works (see Table 1 for details) and pilot trials conducted in our laboratory. The sources, concentrations, and characterization details of the antibodies used are provided in Table 1.

### 4.2 Immunocytochemical procedures

After 72 h of post‐fixation, mouse brains were washed several times with PB and were cut with a vibratome into 30 μm‐thick coronal sections, which were collected serially (in groups of five) in phosphate‐buffered saline (PBS) and stored at 4°C until processing.

#### 4.2.1 Immunoperoxidase Studies

For light microscopic studies, free‐floating sections of mouse brains were pretreated with H_2_O_2_ (1% in PBS, 30 min) to remove endogenous peroxidase, rinsed with PBS, and then incubated for 2 h at room temperature with blocking solution (10% normal donkey serum) and then in primary antibody (BEST1) for 2 h at room temperature and then overnight at 4°C (Table 1). The following day, sections were rinsed three times in PBS and incubated in a solution of blocking buffer containing biotinylated goat anti‐rabbit IgG for one hour at room temperature (Table 1). Sections were subsequently rinsed in PBS, incubated in the avidin‐biotin‐peroxidase complex, washed several times in PBS, and incubated in 3,3′‐diaminobenzidine tetrahydrochloride (DAB; 0.05% in Tris 0.05 M with 0.03% H_2_O_2_). Sections were washed in PB, mounted on gelatin‐coated slides, air‐dried, coverslipped, and finally examined with a Leitz Orthoplan (Wetzlar, Germany) microscope. Method specificity was controlled by either substituting primary antibodies with PBS or by performing a series of immunoperoxidase running using free‐floating sections of BEST1 knockout mouse brains.

#### 4.2.2 Double‐labeling immunofluorescence studies

For double‐labeling studies, free‐floating sections of mouse brains were incubated for 1 h in 10% normal donkey serum (NDS) in PB and then in primary antibodies (BEST1/S100β; BEST1/NeuN; BEST1/IBA1; BEST1/NG2; BEST1/VGLUT1 and BEST/VGAT) for 2 h at room temperature and then overnight at 4°C (Table 1). The next day, samples were incubated for 30 min in 10% NDS in PB and subsequently in a mixture of appropriate secondary fluorescent antibodies (Table 1). They were then mounted, air‐dried, and coverslipped using Vectashield mounting medium (H‐1000; Vector Laboratories, Burlingame, CA). Samples incubated only with the secondary antibody, used as control, revealed no cross‐reactivity. Immunofluorescence images were acquired with a Leica SP2 TCS‐SL confocal microscope (Leica Microsystems, Wetzlar, Germany).

#### 4.2.3 Imaris 3D reconstruction procedure

For 3D reconstruction, *z*‐stack images (8 to 25 μm depth, 0.35 μm step) were acquired with a planapo 63× objective (numerical aperture 1.4, pinhole 1.0, image size 512 × 512 pixels, yielding a frame of 79.35 μm). Signal acquisition was optimized; photomultiplier gain was set so that the brightest pixels were just slightly below saturation and offset such that the darkest pixels were just above zero. To improve the signal/noise ratio, 8–10 frames/image were averaged. Raw LEI files were used for further analysis using Imaris software (v. 10.1, Oxford Instruments, Abingdon‐on‐Thames, UK) as previously described.^20^, ^40^, ^41^ Briefly, after importing each *z*‐stack image into the Imaris arena, the “*FIJI‐bridge*” extension was used to transfer them to FIJI/ImageJ software (v. 1.54f, NIH, USA).^42^ Images were deconvolved using the “*Iterative Deconvolve 3D*” plug‐in as previously described,^43^ with the number of iterations set to 10 based on pilot trials conducted in our lab; all other parameters remained unchanged. Post‐deconvolution, the images were transferred back to Imaris for surface reconstruction. Before reconstruction, the slice view was used to inspect each channel and measure the diameter (in μm) of the largest objects in the field, utilizing the draw tool. A minimum of five measurements per channel were averaged. These average diameters for each target—BEST1, S100β, NeuN, IBA1, NG2, VGLUT1, and VGAT—were applied for “*Background Subtraction*” during surface reconstruction. The “*Lower Voxel Limit*” was adjusted to optimize surface reconstruction for each target. Following surface reconstruction, the filter function was applied to exclude nonspecific background signals, ensuring reliable object‐object colocalization data. The “*Shortest Distance to Surface*” filter was used to isolate BEST1 elements that entirely intersected other reconstructed surfaces. Only BEST1 elements with a distance of 0 μm or less (distance‐filtered BEST1 elements) from other surfaces were considered for further analysis. These distance‐filtered BEST1 elements were then analyzed using the “*Overlapped Volume to Surfaces*” function to assess colocalization with either cellular (S100β, NeuN, IBA1, NG2) or presynaptic (VGLUT1, VGAT) markers. Only the distance‐filtered BEST1 reconstructed elements with an overlapped ratio value included between 80% and 100% (thus, virtually entirely overlapped) with S100β, NeuN, IBA1, NG2, and VGLUT1 or VGAT reconstructed surfaces were considered colocalized and collected for the analyses. Finally, all data were exported to separate Excel files and used for data and statistical analysis.

### 4.3 Statistical analysis

Data distribution was explored using the Shapiro–Wilk normality test to establish whether parametric or nonparametric statistics were appropriate. The homoscedasticity/homogeneity of variance means was examined using Levene's test. Data were analyzed using GraphPad Prism 9 (GraphPad Software, San Diego, CA, USA). Significance was accepted at *p* < 0.05. The statistical design for each analysis is detailed in the corresponding figure legend.

All submitted materials and data adhere to the good publishing practices outlined in the Acta Physiologica guidelines for physiology research.^44^

## 5 CONCLUSIONS

Here, we demonstrated that BEST1 is constitutively expressed in parietal cortical neurons, microglia, and oligodendrocytes. Notably, we showed that neurons represent the second most abundant BEST1^+^ cell type in the cerebral cortex, following astrocytes, and that this channel is localized at both extrasynaptic and synaptic domains. This evidence challenges the astrocentric view of its function, at least at the cortical level. These findings contribute to a broader understanding of BEST1's distribution and its role in controlling GABA and glutamate levels in higher‐order circuitry. However, further studies are crucial to fully unravel BEST1's involvement in tuning the extracellular levels of these neurotransmitters at these cellular domains.

Our current findings raise several questions: (i) Is neuronal BEST1 directly involved in regulating glutamate and GABA tone at the cortical level? (ii) Can presynaptic BEST1 modulate phasic GABA and glutamate transmission? (iii) What is the role of neuronal BEST1 in cortical circuitry, and most importantly, does it affect synaptic plasticity in physiological and pathological conditions? (iv) Does BEST1 expression in microglia and oligodendrocytes impact cortical synaptic transmission in both physiological and pathological conditions?

The current evidence emphasizes distinct roles for this anion channel at the astrocytic level in controlling GABA and glutamate tone across different brain areas and in neurological diseases,^17^, ^18^ underscoring the need for a more comprehensive understanding of its role in different cellular compartments through further research. Indeed, the mechanisms underlying BEST1's function in neurons, microglia, and oligodendrocytes remain largely unexplored. Unraveling these pathways is crucial, as it could reveal novel insights into the regulation of neurotransmission and cellular interactions, potentially unlocking new therapeutic strategies for neurological and psychiatric disorders. These gaps in knowledge highlight an urgent need for future research to fully elucidate BEST1's role in both physiological and pathological conditions.

## AUTHOR CONTRIBUTIONS

**Michael Di Palma:** Conceptualization; methodology; investigation; formal analysis; validation; data curation; visualization; writing—original draft; writing—review and editing. **Wuhyun Koh:** Methodology; validation; writing—review and editing. **C. Justin Lee:** Provided the BEST1 antibody and BEST1 knockout brain samples; validation; writing—review and editing. **Fiorenzo Conti:** Conceptualization; funding acquisition; writing—review and editing; project administration; supervision; resources; visualization.

## FUNDING INFORMATION

This study was supported by funds granted by PRIN 2022 to Fiorenzo Conti.

## CONFLICT OF INTEREST STATEMENT

The authors declare that the research was conducted in the absence of any commercial or financial relationships that could be construed as a potential conflict of interest.

## Data Availability

The raw data supporting the conclusions of this article will be made available by the authors, without undue reservation.
